# Online Lifestyle Support for Adolescents Undergoing Metabolic and Bariatric Surgery: Pilot Prospective Cohort Study

**DOI:** 10.2196/86816

**Published:** 2026-10-05

**Authors:** Sarah E Messiah, Jackson M Francis, Folefac D Atem, Matthew Sunil Mathew, Alicia Wheelington, Maral Misserian, Sitapriya Neti, Rashon Braxton, Bethany R Cartwright, Faisal G Qureshi, Sarah E Barlow, Marlyn A Alicock

**Affiliations:** 1 Peter O’Donnell Jr. School of Public Health, The University of Texas Southwestern Medical Center, 5323 Harry Hines Blvd, Dallas, TX, 75390, United States, 1 3057785309; 2 Department of Pediatrics, University of Texas Southwestern Medical Center, Dallas, TX, United States; 3 Child and Adolescent Population Health Program, Children’s Health System of Texas, Dallas, TX, United States; 4 Children’s Health System of Texas, Dallas, TX, United States; 5 University of Texas Health Science Center at Houston (UTHealth) School of Public Health, Dallas, TX, United States; 6 Department of Psychiatry, University of Texas Southwestern Medical Center, Dallas, TX, United States; 7 School of Allied Health Professions, University of Texas Southwestern Medical Center, Dallas, TX, United States; 8 Touchstone Diabetes Center, University of Texas Southwestern Medical Center, Dallas, TX, United States; 9 Department of Surgery, University of Texas Southwestern Medical Center, Dallas, TX, United States

**Keywords:** adolescents, metabolic and bariatric surgery, lifestyle intervention, weight loss, cardiometabolic

## Abstract

**Background:**

Metabolic and bariatric surgery (MBS) is a safe and effective treatment for adolescents with severe obesity, yet no standardized lifestyle interventions exist to support sustained behavior change after MBS.

**Objective:**

This pilot study assessed the feasibility and acceptability of TeenLyft, an online lifestyle support program for adolescents undergoing MBS, and explored preliminary clinical and safety outcomes to inform the design of a future randomized trial.

**Methods:**

Adolescents aged 13 to 18 years were recruited from a tertiary care MBS center and enrolled in TeenLyft. The study was not powered or designed as a randomized controlled trial but rather as a pilot to guide future randomized controlled trial development. Feasibility domains included recruitment, retention, data completeness, intervention fidelity, and engagement, with predefined progression criteria of ≥80% enrollment, ≥70% retention, ≥70% data completeness, and ≥90% fidelity. Exploratory clinical and safety measures (weight, BMI, and cardiometabolic markers) were collected descriptively to confirm that participation was not associated with harm.

**Results:**

Twenty-nine adolescents (mean age 15.9 years, SD 1.1; n=22, 75.9% female; n=13, 44.8% Hispanic; mean BMI 47.8, SD 7.3 kg/m^2^) were enrolled, representing 145% of the recruitment target (n=20), with 72.4% retention at 6 months. All feasibility progression criteria were met. Descriptive trends showed expected post-MBS reductions in weight and BMI and improvements in blood pressure and hemoglobin A_1c_, with no adverse cardiometabolic effects observed.

**Conclusions:**

TeenLyft demonstrated high feasibility, acceptability, and safety as an adjunct to adolescent MBS care. Findings support progression to a fully powered randomized trial to evaluate long-term effectiveness and sustainability.

## Introduction

Severe obesity in childhood, defined as a BMI ≥120% of the 95th percentile for age and sex or an absolute BMI >35 kg/m^2^, represents the most rapidly increasing form of pediatric obesity in the United States [1]. Among adolescents aged 13 to 19 years, the prevalence of class III obesity rose from 2.1% in 2019 to 2.7% in 2023, equivalent to nearly 1 million youth (*P* value for trend <.001) [2]. Reflecting its growing impact, the American Academy of Pediatrics (AAP) has described pediatric severe obesity as “an epidemic within an epidemic” [3]. This condition contributes to elevated risks for cardiometabolic, hepatic, and renal complications, as well as diminished sleep quality and mental health, ultimately lowering health-related quality of life [3-5]. In addition, obesity in adolescence strongly predicts adult obesity and is linked to increased rates of asthma, arthritis, metabolic dysfunction, psychological distress, and at least 13 forms of cancer, contributing to reduced life expectancy [6-10].

Although behavioral lifestyle programs are foundational to pediatric obesity treatment, evidence indicates that such programs alone rarely produce sustained or clinically meaningful weight loss in adolescents with severe obesity [11-14]. In contrast, metabolic and bariatric surgery (MBS) has been shown to be both effective and safe for this population [15-18]. The 2019 AAP clinical practice statement recommends a family-centered, nonstigmatizing approach to obesity treatment that includes behavioral and lifestyle interventions, pharmacotherapy beginning around age 12 years, and MBS as an option for adolescents with severe obesity from approximately age 13 years when clinically appropriate [3,19]. To promote equitable access, the AAP further emphasizes that health care providers should inform families about surgical options and provide comprehensive preoperative and postoperative support [19]. Consistent with this guidance, national data show a steady rise in MBS use among US adolescents [2,20]. The treatment landscape for adolescent obesity continues to evolve with the approval of glucagon-like peptide-1 receptor agonists for pediatric obesity treatment. Although these medications have expanded therapeutic options for adolescents with severe obesity, MBS remains the most effective intervention for achieving substantial and sustained weight loss among appropriately selected youth [15-18]. Accordingly, there remains an ongoing need for strategies that support long-term behavioral adherence following surgery.

Despite this progress, there remains a critical gap in standardized, age-appropriate lifestyle interventions designed to accompany and sustain the behavioral changes required for long-term success after MBS [21,22]. To address this gap, we developed TeenLyft, a tailored online lifestyle support program grounded in the principles of the Diabetes Prevention Program Group Lifestyle Balance (DPP-GLB) model [23-27]. Prior DPP-GLB implementations have demonstrated improvements in BMI and cardiometabolic markers in adult populations [28]. The adaptation process for TeenLyft incorporated adolescent and caregiver input to prioritize relevant content and delivery methods. Core curriculum topics included caloric balance, nutrition, physical activity, and strategies for overcoming setbacks. Formative research also identified key motivational factors (eg, health improvement and family encouragement) and barriers (eg, fear of complications and lack of peer support) that informed the intervention design [29].

The present study reported findings from the pilot evaluation of TeenLyft among adolescent MBS recipients. Specifically, this analysis examined feasibility domains including recruitment, retention, data completeness, intervention fidelity, and participant engagement, as well as exploratory safety indicators (eg, weight, BMI, and cardiometabolic markers) to ensure the program was not associated with harm. These pilot data were collected to inform the design, progression criteria, and outcome selection for a future randomized controlled trial (RCT).

## Methods

### Procedures

In 2021, we were awarded funding from the Eunice Kennedy Shriver (NCT05393570) to develop and evaluate a lifestyle support program tailored for adolescents undergoing MBS. The study used a mixed methods, proof-of-concept framework to guide intervention development and pilot testing [30]. Between January 2023 and March 2024, participants were recruited from a multidisciplinary adolescent MBS clinic offering integrated psychological and nutritional care. The TeenLyft intervention content was codeveloped with input from adolescents and caregivers, drawing on principles of the DPP-GLB curriculum.

In brief, TeenLyft was a 6-month behavioral lifestyle support intervention adapted from the DPP-GLB curriculum and tailored for adolescents undergoing MBS. Guided by social cognitive theory and formative work with adolescents and caregivers, TeenLyft addressed healthy eating, physical activity, stress and emotion management, motivation, and strategies for maintaining weight-related behavior change following surgery. Content was delivered through brief online video modules distributed through YouTube (Alphabet Inc) and Facebook (Meta Platforms). A total of 86 videos were developed, including preoperative and postoperative content, with videos averaging approximately 2 to 5 minutes in length. Additional details regarding intervention development and adaptation have been published previously [29].

### Ethical Considerations

Human subjects approval was obtained prior to study initiation from the University of Texas Health Science Center at Houston Institutional Review Board (IRB; HSC-SPH-19‐0406), with the University of Texas Southwestern Medical Center and the Children’s Health System of Texas participating under IRB reliance agreements. Parental consent and adolescent assent were secured in accordance with ethical standards. Although consent materials were available in English and Spanish, all participants elected to complete the study in English. All study procedures were conducted in accordance with the ethical standards of the institutional review board and the Declaration of Helsinki.

### Study Design

This investigation used a quasiexperimental, prospective cohort design without a control group or random assignment, consistent with the intent of a pilot feasibility study. The primary objective was to evaluate feasibility and acceptability outcomes including recruitment, retention, data completeness, intervention fidelity, and participant engagement to inform the design and implementation of a future fully powered RCT. Given this feasibility focus, no control group was included, and statistical analyses were descriptive in nature. Reporting followed the TREND (Transparent Reporting of Evaluations with Nonrandomized Designs) guidelines, which complement the CONSORT (Consolidated Standards of Reporting Trials) extension for pilot and feasibility trials by emphasizing transparent reporting of design, conduct, and feasibility outcomes for nonrandomized studies. Because this was a single-arm feasibility study without randomization or treatment allocation, participants, study staff, and outcome assessors were not blinded. Clinical outcome measures were obtained from routinely collected electronic health record data using standardized clinical procedures.

The study was guided by the RE-AIM (reach, effectiveness, adoption, implementation, and maintenance) framework. RE-AIM informed the selection of feasibility outcomes by focusing on participant reach (enrollment), implementation (intervention fidelity), and maintenance (retention and follow-up completion). Consistent with the goals of a pilot study, recruitment, retention, data completeness, intervention fidelity, and participant engagement were selected as primary feasibility indicators to inform progression to a future randomized trial. Exploratory clinical outcomes, including anthropometric and cardiometabolic measures, were collected to evaluate safety and identify candidate end points for future efficacy testing and are reported elsewhere [30].

### Participants

Adolescents were recruited from the adolescent MBS program at Children’s Health System of Texas. Eligibility criteria included (1) age 13 to 18 years, (2) meeting National Institutes of Health criteria for adolescent MBS [31], (3) ability to communicate in English or Spanish, and (4) access to study materials through the internet or a smartphone. Participants were required to complete the standard multidisciplinary clinical evaluation process for MBS, including psychological clearance. Exclusion criteria included adolescents who were not medically eligible for MBS, declined participation, or had diabetes requiring mealtime insulin. Eligibility criteria were intentionally broad to reflect real-world clinical practice and support evaluation of intervention feasibility and acceptability in a representative adolescent MBS population. All participants underwent laparoscopic sleeve gastrectomy. Roux-en-Y gastric bypass was not performed among adolescents treated within the study program during the enrollment period; therefore, no participants undergoing gastric bypass were available for inclusion.

### Feasibility Measures

Feasibility outcomes were the primary end points of this pilot study. Enrollment counts (consented participants) were tracked against a target of 20 participants to assess recruitment success. Retention rates at 3- and 6-month follow-up visits were recorded to evaluate ongoing engagement. Data completeness for anthropometric and laboratory measures was used to assess the feasibility of data collection processes. Online platform analytics (YouTube and Facebook) quantified module views and average watch times during the 6-month intervention. Fidelity was monitored through delivery checklists ensuring adherence to planned content and format, and qualitative notes captured any technology-related barriers or participant burden.

Feasibility was defined a priori as achieving ≥80% of the recruitment target, ≥70% retention by 6 months, ≥70% data completeness, and ≥90% fidelity to planned intervention delivery. Outcome assessments were obtained at baseline (before MBS) and during routine clinical follow-up visits approximately 3 and 6 months following surgery. Anthropometric and cardiometabolic measures were extracted from the electronic health record as part of standard clinical care.

### Exploratory Clinical and Safety Measures

Although not powered for hypothesis testing, exploratory outcomes were collected to ensure the intervention was not associated with harm and to guide end point selection for future trials. Measures included excess weight loss, BMI (raw, percentile, and *z* score), and cardiometabolic indicators (systolic and diastolic blood pressure, hemoglobin A_1c_ (HbA_1c_), fasting glucose, and lipid profiles).

### Covariates

Age, sex, race, ethnicity, and social determinants of health (SDOH) were treated as covariates. SDOH variables were derived from a standardized health system screener comprising 13 questions spanning key domains including health literacy, social support, food security, transportation access, financial stability, housing conditions, and tobacco exposure [31,32]. The screener aligns with the SDOH priority areas outlined in Healthy People 2030 [33]. Specifically, food and housing insecurity correspond to the economic stability domain; tobacco use, transportation, and access to utilities and the internet align with neighborhood and built environment; and social support and health literacy map to the social and community context and health care access and quality domains [34]. The instrument incorporates validated items, such as the hunger vital sign for food insecurity, adapted for pediatric and family use [35].

### Statistical Analysis

Feasibility outcomes were summarized descriptively to assess recruitment, retention, data completeness, intervention fidelity, and participant engagement. These metrics were compared against prespecified progression criteria to determine whether the study design and procedures were suitable for a future RCT.

For exploratory purposes, descriptive analyses were used to summarize the study sample’s baseline demographic, clinical, and SDOH characteristics. Continuous variables are presented as means and SDs, and categorical variables as frequencies and percentages. Changes in anthropometric and cardiometabolic indicators at 3 and 6 months after MBS were described using summary statistics and graphical trends to estimate potential effect sizes and to confirm the absence of adverse outcomes.

Although this pilot study was not powered for hypothesis testing, exploratory comparisons were conducted to examine patterns in excess weight loss by participant characteristics. These analyses included nonparametric methods (Wilcoxon signed-rank and Fisher exact tests) and random-effects models for repeated measures to explore within-participant changes over time. Results were interpreted descriptively to guide selection of candidate outcome measures for a future trial rather than to infer efficacy. All analyses were performed using SAS (version 9.4; SAS Institute).

## Results

### Participant Characteristics

Summary descriptive statistics are available in Table 1.

**Table 1.** Participant characteristics before metabolic and bariatric surgery (N=29).

Descriptive characteristics	Participants
Age (y), mean (SD)	15.9 (1.1)
Age (y), n (%)	
13‐16	19 (65.5)
17‐18	10 (34.5)
Sex, n (%)	
Female	22 (75.9)
Male	7 (24.1)
Race and ethnicity, n (%)	
Non-Hispanic White	4 (13.8)
Non-Hispanic Black	11 (37.9)
Hispanic	13 (44.8)
Other	1 (3.4)
Weight (kg), mean (SD)	135.0 (22.1)
BMI (kg/m^2^), mean (SD; range)	47.8 (7.3; 36.9‐62.4)
Social determinants of health (positive), n (%)	
Food insecurity	4 (13.8)
Health literacy challenges	5 (17.2)
Inadequate social support	16 (55.2)
Transportation insecurity	3 (10.3)
Financial insecurity	5 (17.2)
Housing insecurity	1 (3.4)

### Feasibility Outcomes

A total of 29 adolescents enrolled in TeenLyft, exceeding the planned sample of 20 and achieving 145% of the recruitment target. Retention at 6 months was 72.4% (21/29), with these 21 participants completing all scheduled follow-up assessments, clinical appointments, and laboratory measures. Most attrition reflected missed follow-up visits or inability to complete scheduled assessments rather than adverse events or concerns related to the intervention. Data completeness exceeded the 70% threshold for anthropometric and biomarker collection. The intervention was delivered with 100% fidelity to planned content and schedule. Across both online platforms (YouTube and Facebook), 86 session modules were delivered, garnering 520 total views and an average watch time of 3.38 minutes per module, indicating consistent participant engagement. No major technology barriers or participant burden were reported. Informal feedback collected during clinical visits indicated that participants found the modules easy to navigate and relevant to their postoperative needs. All feasibility progression criteria were achieved (Table 2).

**Table 2.** Feasibility outcomes and progression criteria for the TeenLyft pilot study.

Feasibility domain	Definition or metric	Prespecified target	Observed outcome	Target met?
Recruitment	Number and percent of participants enrolled compared to target (n=20 planned)	≥80% of target enrollment	29 enrolled (145% of target)	Yes
Retention	Participants completing 6-month follow-up assessments	≥70%	72.4% (21 of 29 enrolled participants); >100% (21 of planned 20 participants)	Yes
Data completeness	Participants with complete anthropometric and laboratory data through 6 months	≥70%	72.4% (21 of 29 enrolled participants); >100% (21 of planned 20 participants)	Yes
Intervention fidelity	Sessions delivered as planned (video content and schedule)	≥90%	100% delivered as planned	Yes
Participant engagement	Online module views and average watch time (YouTube and Facebook)	Descriptive only	520 total views; 3.38-min average watch time	N/A^a^
Acceptability	Informal feedback on ease of use and relevance	Qualitative assessment	Participants reported that the content was easy to navigate and relevant	Positive

a N/A: not applicable.

### Exploratory Clinical and Safety Outcomes

Exploratory analyses of weight and cardiometabolic indicators were conducted to confirm that the intervention was not associated with harm and to estimate potential effect sizes for future studies. From baseline to 6 months after MBS, participants exhibited expected postoperative improvements, including decreases in mean weight from 136.1 (SD 28.0) kg to 95.7 (SD 15.9) kg and mean BMI from 48.3 (SD 8.3) kg/m² to 33.3 (SD 4.5) kg/m², as well as decreases in BMI percentile and *z* score. Systolic blood pressure and HbA_1c_ showed modest decreases, while diastolic blood pressure, fasting glucose, and lipid profiles remained stable (Table 3). These trends are consistent with anticipated post-MBS trajectories and suggest that participation in TeenLyft did not adversely affect expected surgical outcomes.

**Table 3.** Anthropometric and cardiometabolic biomarker change from before metabolic and bariatric surgery (MBS) to 3 and 6 months after MBS (n=21).

Outcome	Before MBS, mean (SD)	3 months after MBS, mean (SD)	6 months after MBS, mean (SD)	*P* value
Anthropometric measures				
Weight (kg)	136.1 (28.0)	110.3 (15.7)	95.7 (15.9)	<.001^a^
BMI (kg/m^2^)	48.3 (8.3)	38.4 (5.6)	33.3 (4.5)	<.001
BMI percentile	99.7 (0.7)	98.5 (1.5)	96.5 (2.8)	<.001^b^
BMI *z* score	3.7 (1.2)	2.4 (0.5)	1.9 (0.4)	<.001^a^
Cardiometabolic outcomes^c^				
Systolic blood pressure (mm Hg)	125.4 (14.3)	119.2 (11.8)	117.6 (13.6)	.04
Diastolic blood pressure (mm Hg)	71.4 (10.3)	69.1 (11.2)	66.4 (8.6)	.17
Glucose (mg/dL)	99.5 (35.4)	86.3 (5.5)	88.0 (7.6)	.31
Hemoglobin A_1c_ (%)	5.7 (1.2)	4.9 (0.1)	5.1 (0.3)	.049
Total cholesterol (mg/dL)	151.2 (29.6)	0^d^	146.8 (23.7)	.59
High-density lipoprotein cholesterol (mg/dL)	39.6 (7.1)	0	43.2 (9.0)	.10
Low-density lipoprotein cholesterol (mg/dL)	90.4 (25.7)	0	86.2 (24.5)	.57
Triglycerides (mg/dL)	106.8 (49.1)	0	87.4 (21.1)	.12

a Multiple comparison testing using Tukey approach showed statistically significant differences between before MBS and 3 months after MBS and before MBS and 6 months after MBS, but no statistical difference between 3 and 6 months after MBS.

b Tukey comparison showed statistically significant differences across all time points.

c Lipid profiles were only collected at 6 months after MBS.

d Three-month lipid results are not summarized because data were available for only one participant, precluding calculation of an SD.

### Exploratory Patterns in Excess Weight Loss

Although the study was not powered for hypothesis testing, exploratory comparisons were conducted to describe patterns in excess weight loss (EWL) by participant characteristics. Adolescents achieving EWL >50% tended to be older and more likely to be female compared with those with EWL ≤50%. No clear differences were observed by presurgery BMI or SDOH variables (Table 4). These patterns are provided descriptively to guide refinement of subgroup analyses and sample size planning for a future randomized trial.

**Table 4.** Comparison of excess weight loss (EWL) at 6 months among adolescents who participated in TeenLyft^a^.

Variables	EWL >50% (n=9)	EWL ≤50% (n=12)	*P* value^b^
Age (y), mean (SD)	16.8 (0.7)	15.3 (1.1)	.008
Sex, n (%)			
Female	4 (44.4)	0 (0)	.006
Male	5 (55.6)	12 (100)	
Race and ethnicity, n (%)			.06
Hispanic or Latino	7 (77.8)	7 (58.3)	
Non-Hispanic Black	0 (0)	4 (33.3)	
Non-Hispanic White	2 (22.2)	0 (0)	
Other	0 (0)	1 (8.3)	
Presurgery BMI, mean (SD)	48.9 (7.6)	44.1 (5.3)	.19
Food insecurity, n (%)			.40
No	1 (33.3)	3 (100)	
Yes	2 (66.7)	0 (0)	

a This pilot study was not powered for hypothesis testing; all comparisons are descriptive and intended to inform outcome selection for future trials.

b Comparisons between groups (EWL >50% vs ≤50%) were evaluated using Wilcoxon signed-rank and Fisher exact tests, as appropriate.

## Discussion

### Principal Findings

This pilot study primarily evaluated the feasibility, acceptability, and safety of implementing TeenLyft, a virtual lifestyle support intervention, for adolescents undergoing MBS. The study demonstrated high recruitment, retention, and engagement, with all predefined feasibility progression criteria achieved. Recruitment feasibility was particularly strong, with enrollment exceeding the funded target (29 vs 20 participants), suggesting both high interest and successful clinic integration. This overenrollment further supports the feasibility of scaling up TeenLyft for a larger randomized trial. Participants reported the program to be accessible, relevant, and easy to use, and the intervention was delivered with full fidelity and no technological barriers. Collectively, these findings confirm that TeenLyft is a feasible and acceptable adjunct to standard adolescent MBS care and can be scaled for evaluation in a larger randomized trial.

Exploratory clinical and safety outcomes were collected to verify that participation in TeenLyft was not associated with adverse effects and to generate preliminary effect-size estimates for future research. As expected, participants experienced reductions in weight, BMI, and systolic blood pressure, along with modest improvements in HbA_1c_ over the first 6 months after surgery. Fasting glucose and lipid profiles remained stable. These trends align with previously reported outcomes for adolescent MBS cohorts [36-46], suggesting that the addition of TeenLyft did not negatively influence expected surgical results. Importantly, these findings support the safety of integrating structured behavioral support into postoperative care.

Although exploratory patterns suggested that older adolescents experienced greater EWL than younger participants and that male participants exhibited smaller reductions, the small sample size precludes firm conclusions. These patterns are consistent with prior literature indicating potential age- and sex-related differences in short-term postoperative outcomes [37-41]. Similarly, while SDOH were not strongly associated with weight outcomes, trends such as higher food insecurity among those with greater EWL highlight complex resilience factors that warrant exploration in future work [47-50].

Psychosocial outcomes were not included as formal quantitative end points in this pilot feasibility study, but psychosocial experiences have been examined within the broader TeenLyft research program [51]. Prior qualitative work identified the importance of motivation, social support, body image, weight stigma, and mental health throughout the adolescent MBS journey. Consistent with those findings, participants in the present study described perceived improvements in self-confidence, body image, motivation, and emotional well-being following surgery and participation in TeenLyft [51,52]. Future randomized trials should incorporate validated psychosocial measures to more comprehensively evaluate both benefits and potential unintended consequences of behavioral support interventions following adolescent MBS.

These feasibility findings reinforce the value of integrating digital, flexible support into adolescent MBS pathways. Online engagement through TeenLyft modules demonstrates that adolescents are willing to participate in brief, accessible, and self-directed educational content alongside clinical care. This approach may help maintain continuity of behavioral reinforcement between visits and facilitate equitable access to postoperative support, key priorities emphasized in the AAP’s guidance on comprehensive obesity treatment [3,19].

### Limitations and Future Directions

This study has several limitations. The small sample size, single-site design, and 6-month follow-up limit generalizability and preclude assessment of long-term outcomes. The relatively short follow-up period is particularly important given that obesity is a chronic disease requiring long-term management. While the first 6 months following MBS are characterized by rapid weight loss and high clinical engagement, longer-term outcomes may be influenced by changes in adherence, psychosocial factors, nutritional challenges, use of antiobesity medications, or the need for additional surgical or medical interventions. Future studies should evaluate whether behavioral support interventions such as TeenLyft contribute to sustained weight management and health outcomes beyond the early postoperative period. Additionally, the absence of a control group was intentional and appropriate for a feasibility study but prevents direct evaluation of comparative effectiveness. Nonetheless, observed weight and cardiometabolic outcomes were consistent with published benchmarks for MBS in adolescents [42-47], indicating that TeenLyft did not compromise expected clinical benefits. Future studies should include a larger, more diverse sample, extend follow-up duration, and incorporate quantitative engagement metrics to assess dose-response relationships between participation and outcomes. A randomized design comparing MBS alone versus MBS plus TeenLyft will be essential to determine the program’s added value beyond feasibility and safety.

### Conclusions

TeenLyft demonstrated high feasibility, acceptability, and safety as a virtual adjunct to adolescent MBS care. The program achieved or exceeded all predefined feasibility criteria and showed no evidence of harm to expected postoperative outcomes. These findings provide critical groundwork for a fully powered RCT to assess the effectiveness, cost, and long-term sustainability of integrating structured, technology-based lifestyle support into standard adolescent MBS care.

## Abbreviations

AAP: American Academy of Pediatrics
CONSORT: Consolidated Standards of Reporting Trials
DPP-GLB: Diabetes Prevention Program Group Lifestyle Balance
EWL: excess weight loss
HbA_1c_: hemoglobin A_1c_
MBS: metabolic and bariatric surgery
RCT: randomized controlled trial
RE-AIM: reach, effectiveness, adoption, implementation, and maintenance
SDOH: social determinants of health
TREND: Transparent Reporting of Evaluations with Nonrandomized Designs
